# Effect of Different Slow-Release Urea on the Production Performance, Rumen Fermentation, and Blood Parameter of Angus Heifer

**DOI:** 10.3390/ani14162296

**Published:** 2024-08-07

**Authors:** Caiyun Fan, Hongguang Li, Shuaihong Li, Gang Zhong, Wenbin Jia, Zhao Zhuo, Yanfeng Xue, Anne F. Koontz, Jianbo Cheng

**Affiliations:** 1 College of Animal Science and Technology, Anhui Agricultural University, Hefei 230036, China; fancaiyunnmgbt@163.com (C.F.); 19808628817@163.com (H.L.); shuaishuaipianer@163.com (S.L.); zhuozhao90@163.com (Z.Z.); xueyanfeng1990@163.com (Y.X.); 2 Zhonghe Hengrui (Guizhou) Co., Ltd., Bijie 551600, China; mengyuanzhonggang@163.com; 3 Beijing Alltech Biological Products Co., Ltd., LD01/0702-0703, Liangmaqiao (DRC) Diplomatic Office Building, No. 19 Orient East Road, Chaoyang District, Beijing 100600, China; wejia@alltech.com; 4 Center for Animal Nutrigenomics and Applied Animal Nutrition, Alltech Inc., Nicholasville, KY 40356, USA; akoontz@alltech.com

**Keywords:** polymer-coated urea, gelatinized starch urea, heifers, rumen microbiota

## Abstract

**Simple Summary:**

Slow-release urea can slow down the degradation rate of urea in the rumen, improving nitrogen utilization efficiency and promoting the growth of rumen microorganisms. However, different slow-release urea may have variance functions. This study used two kinds slow-release urea, polymer-coated urea and gelatinized starch urea, to evaluate the effects on Angus heifers. We found that polymer-coated urea was able to improve the feed efficiency of cattle, and it also increased the daily gain. In rumen, adding polymer-coated urea increased the content of volatile fatty acids like acetate and propionate. In addition, polymer-coated urea increased the relative abundance of some friendly bacteria in the rumen, like *Paraprevotella*. Thus, we believe that polymer-coated urea can improve the production performance of Angus heifers and bring about more economic benefits.

**Abstract:**

This study investigated the effect of replacing part of the dietary soybean meal with either polymer-coated urea or gelatinized starch urea on the production performance, blood indexes, and ruminal fermentation of Angus heifers. A total of 210 purebred Angus cattle (BW = 314.26 kg) were divided into three groups: the no urea group (CON), the polymer-coated urea group (PCU), and the gelatinized starch urea group (GSU); 20 g/kg polymer-coated urea or 25 g/kg gelatinized starch urea was used to replace part of soybean meal in the concentrate feed, according to the principle of isometabolic energy and isonitrogenous. The result showed that the PCU group had higher ADG and ADF apparent digestibility, while it had a lower feed–weight ratio. On the 86th day, the serum albumin (ALB) content in the PCU group was significantly higher than that in the CON group. In rumen, compared with the CON group, the contents of acetic acid and total volatile fatty acid were significantly higher in the PCU group, whereas butyric acid and propionic acid were significantly higher in the PCU group and GSU group. Ruminal bacterial diversity analysis found that the abundance of *Firmicutes* was higher in the PCU group at the phylum level, and an inverse result was observed in *Bacteroidetes*. The abundance of *Paraprevotella* was higher in the PCU group, whereas higher abundance of *Prevotella* was found in the GSU group at the genus level. These results indicate that slow-release urea can replace part of soybean meal in the diet, and the amount of substitution in this trial had no diverse effect on the performance of Angus heifers.

## 1. Introduction

With the rapid development of the ruminant animal breeding industry, animal production in China is facing serious challenges including resource constraints, environmental requirements for sustainable development, and growing demand for animal protein. Dietary protein plays an important role in ruminant nutrition, providing amino acids and nitrogen sources for rumen microbial protein production. However, it usually comes from expensive feed, such as soybean meal [1]. In China, the shortage of protein feed resources has become a neck problem in our breeding industry, which has seriously restricted the healthy progress of ruminant breeding. Therefore, the development and utilization of protein feed substitutes, including non-protein nitrogen, to reduce the utilization of soybean meal is one of the most important and difficult topics at present [2].

Urea is a nitrogenous substance excreted by mammals, and the urea cycle in ruminants is unique to their nutritional metabolism. Due to its low cost, good availability, and easy handling and application, it is an important protein supplement source for ruminants [1]. Urea is rapidly hydrolyzed into ammonia by urease secreted by bacteria after entering the rumen, and then it is synthesized into microbial protein for use by the organism. However, the rate of hydrolysis of urea in the rumen is much faster than the rate of ammonia utilization by rumen microorganisms, thus causing the accumulation of ammonia in the rumen and large excretion of ammonia in the urine, which could pollute the environment and waste nitrogen source [3]. Therefore, synchronizing N supply with microbial requirements in the rumen is an essential nutritional strategy to improve rumen energy and N utilization. Slow-release urea is an efficient non-protein nitrogen [4]. The main slow-release urea products used in ruminant are polymer-coated urea and gelatinized starch urea, both of which have different slow-release protocols and large price differences (1040 USD/ton in polymer-coated urea and 885 USD/ton in gelatinized starch urea). Both of them in fattening cattle have been shown to improve animal performance without affecting rumen fermentation, but their effects on rumen microorganisms are rarely reported [1,4]. Therefore, the purpose of this experiment was to study polymer-coated urea and gelatinized starch urea in the production of Angus heifers in relation to production performance, nutrient digestibility, blood biochemical indexes, rumen fermentation indexes, and rumen microbial flora.

## 2. Materials and Methods

### 2.1. Animals, Diets, and Treatments

The experiment was conducted at the second breeding farm of Zhonghe Heng Rui Co., Ltd. (Guizhou, China), from 13 February to 19 May 2020. A total of 210 pure-bred Angus cows were randomly divided into three groups according to the body weight (314.26 kg) and month age (12.96): the control group was fed the basal diet without urea (CON); the polymer-coated urea group was fed 20 g/kg polymer-coated urea instead of partial soybean meal in a concentrate diet (PCU); and the gelatinized starch urea group that was fed 25 g/kg gelatinized starch urea to partially replace soybean meal in the concentrate (GSU). Notably, the polymer-coated urea used in this experiment was provided by Alltech (Nicholasville, KY, USA), namely, SRU (Optigen^®^). It is composed of urea evenly coated with a semi-permeable vegetable fat matrix containing 880 g/kg urea (410 g/kg N, 2560 g/kg crude protein (CP) and 110–120 g/kg fat. Gelatinized starch urea was purchased from Hebei Xingmu Agricultural Products Technology Co., Ltd. (Shijiazhuang, China); raw materials are composed of urea and sodium chloride; and the carrier was corn and bentonite, about 0.3. The measured content of N was 325 g/kg. The polymer-coated urea and the gelatinized starch urea were added in meal form to the complete feed and thoroughly mixed. Diets contained no antibiotics or other growth promoters. The basal diet was prepared according to the beef cattle nutrition NRC (2016) [5]. The experimental diets were formulated to be of equal energy and nitrogen. The dietary composition and nutritional level of specific total mixed ration (TMR) are shown in Table 1, and the composition and nutritional level of concentrate supplements are shown in Table 2.

The experimental cattle were fed separately by group, and the experimental diets were all fed in the form of TMR, twice a day at 9:00 and 16:00. Cattle were allowed ad libitum access to both the mash feed and straw. Within each barn, cattle had free access to a water tank, which was cleaned once every two days, to ensure clean water quality. A loader was used to clean up cattle manure twice a week, and the barn was disinfected with a spray every afternoon. Cattle were individually fed the basal diet for 1 week prior to being switched to the assigned treatments for the 12 week experimental period.

### 2.2. Dissolution of Slow-Release Urea

Urea release from the slow-release urea materials was determined by the methods of Lang (2015) and Pan and Zhang (2018) [6,7]. A total of 3.00 g paradimethylaminobenzaldehyde (PDAB) was dissolved in 150 mL anhydrous ethanol, and then 15 mL concentrated hydrochloric acid was added and mixed well. A total of 0.1250 g pure urea was accurately weighed, dissolved in a 250 mL volumetric flask, and diluted with distilled water to scale; the solution of urea content was 0.5000 g. We took the appropriate amount of 0.5000 mg/mL urea standard solution, with reagent blank as the reference, under the condition of the same dosage of chromogenic agent and chromogenic time change wavelength, determining the absorbance.

We accurately absorbed 0.5000 mg/mL urea standard solution 0.00, 1.00, 2.00, 3.00, 4.00, 5.00, and 6.00 mL in a 25 mL colorimetric tube with the corresponding numbers, and then we accurately added chromogenic agent (10 mL), diluted with distilled water to scale, fully shaken. After the bubbles disappeared for 20 min, the measurement was carried out by spectrophotometer at the maximum wavelength using a 1 cm cuvette with the blank, without urea standard solution as a reference. Then, in vitro dissolution experiments were performed.

Three replicate samples each of 4.47 g polymer-coated urea or 5.63 g gelatinized starch urea were accurately weighed and placed into 750 mL of phosphate buffer solution at pH 6.8, shaken at 38 °C and 100 r/min, and then it was used to simulate the rumen environment. Samples were taken at 1 h, 2 h, 3 h, 4 h, 6 h, and 8 h, and absorbance was measured by spectrophotometer. The release amount of urea at each time point was calculated according to the change of absorbance.

### 2.3. Growth Performance

All cattle were weighed before morning feeding at the beginning of the trial period and once every 30 days. The feed intake of test cattle was recorded daily. The recorded data were used to calculate the average feed intake, average daily gain, and feed to gain.

### 2.4. Nutrient Digestibility

A digestion test was conducted in the last week of the trial, and six cows were selected from each group, with feed and fecal samples collected for three consecutive days. About 500 g of TMR samples were collected by the five-point sampling method after each feeding during the digestion test. Fecal samples were collected through the rectum every 6 h, and about 200 g were collected each time, of which 100 g was nitrogen-fixed with 0.1 hydrochloric acid. We mixed them separately, and then they were frozen at −20 °C. Feed and fecal samples were dried overnight (or until dry) in a conventional drying oven at 55 °C. Dry samples were ground through a 1 mm screen using a pulverizing mill. Feed and fecal samples were analyzed individually for dry matter (DM), crude protein (CP), ether extract (EE), and ash [8]. Sequential analyses of neutral detergent fiber (NDF) and acid detergent fiber (ADF) were determined by the procedure of Van Soest [9]. The acid-insoluble ash (AIA) was used as an internal marker to determine the apparent digestibility of nutrients. TMR, orts, and fecal samples were analyzed according to the procedures by AOAC (2000) [10]. The apparent digestibility of nutrients was calculated as follows: Apparent Digestibility of Nutrients = [1 − (Ad × Nf)/(Af × Nd)] × 100, where Ad = AIA in the diets (g/kg); Af = AIA in the feces (g/kg); Nd = the concentration of a nutrient in the diet (g/kg); Nf = the concentration of a nutrient in the feces (g/kg).

### 2.5. Biochemical Parameters

Ten cattle were selected from each group, and on the 49th and 86th days of the experimental period, 10 mL blood samples were collected from the anterior jugular vein in vacuum tubes with and without anticoagulant. The blood in the anticoagulant tube was fully mixed with the anticoagulant, while the blood in the non-anticoagulant tubes was not mixed. After the supernatant was separated, the blood was centrifuged (3500 r/min, 10 min). The serum and plasma were separated into 1.5 mL tubes, stored at −20 °C, and transferred back to the laboratory and stored at −80 °C. The blood plasma samples were analyzed to determine the concentration of total protein (TP), albumin (ALB), globulin (GLOB), alanine aminotransferase (ALT), aspartate aminotransferase (AST), alkaline phosphatase (ALP), glucose (GLU), total cholesterol (TC), blood urea-N (BUN), and blood ammonia (AN). These biochemical indexes were measured by automatic biochemical analyzer (Beckman AU5800, Beckman Coulter, Inc., Brea, CA, USA) according to the instructions of the corresponding detection kits (Zhongsheng North Control Biological Technology Co., Ltd., Beijing, China), apart from the serum total cholesterol detection kit purchased from Hydrocephalus Medical Co., Ltd., Beijing, China.

### 2.6. Ruminal Fermentation

On the 86th day of the trial period, ten cattle in each group were selected for rumen fluid collection two hours after the morning feeding. Rumen fluid was collected by ruminal sampler (Klibo Animal Husbandry Technology Co., Ltd., Wuhan, China). The sampling catheter was inserted into the rumen through the mouth and esophagus. The first 100 mL of ruminal fluid was collected and was discarded. Another 200 mL of ruminal fluid was collected and filtered through four layers of gauze, and a subsample was stored in a 10 mL centrifuge tube at −20 °C. At the same time, the pH of ruminal fluid before filtering was measured with a pH meter (Shanghai Youke Instrumentation Co., Ltd., Shanghai, China). The samples were taken back to the laboratory and transferred to a −80 °C freezer. The total microbial protein (MCP) in rumen fluid was detected by the Coomassie brilliant blue method, and the concentration of ammonia nitrogen was measured [11,12]. VFA were determined using high-performance liquid chromatography [13].

### 2.7. DNA Extraction and 16S rRNA Pyrosequencing

Three animals from each group of similar age and weight were selected for a second set of rumen fluid sampling following the procedures above. After storage at −80 °C, these samples were sent to the sequencing department of Sheng Gong Biotech Co., Ltd. (Shanghai, China), for sequencing. The main procedures were sample DNA extraction, library construction, and sequencing. Then, we evaluated the quality of extracted genomic DNA by 1% agarose gel electrophoresis. The amplified primers were 338F (5′-ACTCCTACGGGAG-GCAG-3′) and 806R (5′-GGACTACHVGGGT-WTCTAAT-3′) for the V3–V4 regions of the bacterial 16S rDNA gene. The PCR procedure included 3 min of denaturation at 95 °C (1 cycle), then 30 s of denaturation at 95 °C (27 cycle), annealing at 55 °C for 30 s, and elongating at 72 °C for 45 s, with a final extension at 72 °C for 10 min. PCR reactions were performed in triplicate, and the PCR products were extracted from a 2% agarose gel electrophoresis. Then, the AxyPrep DNA gel recovery kit (Axygen Scientific Inc., Union City, CA, USA) was used to purify it. Finally, the amplicons were used for high-throughput sequencing using the Illumina MISeq platform (Illumina, San Diego, CA, USA).

### 2.8. Statistical Analysis

All the original data obtained in the experiment were processed by Excel 2007. SPSS 22.0 statistical software was used for one-way analysis of variance (ANOVA) of the data. Duncan’s method was used for multiple comparisons between groups. Significant differences were declared at *p* < 0.05. Differences at 0.05 < *p* ≤ 0.10 were considered as a trend toward significance.

## 3. Results

### 3.1. Urea Dissolution Profiles

**Figure 1:**
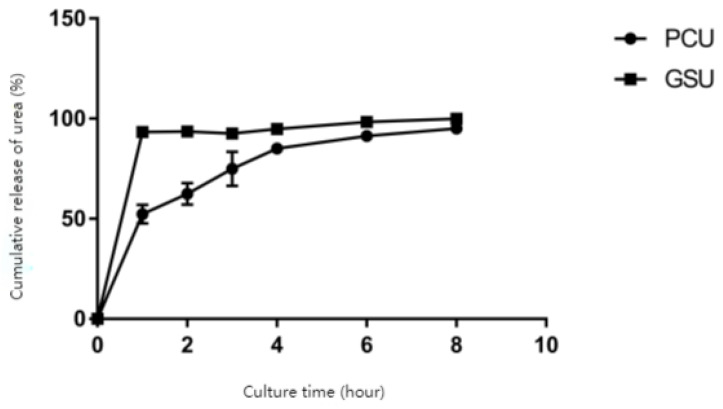
Dissolution of polymer-coated urea and gelatinized starch urea.

The two slow-release urea forms in this study had different dissolution profiles in phosphate buffer solution at pH 6.8 (Figure 1). After two hours, the release rate of the urea in polymer-coated urea was 624.30 g/kg, while the rate of the urea in gelatinized starch urea was 935.30 g/kg. After 8h of digestion, the dissolve rate of urea in polymer-coated urea was 951.00 g/kg, and the rate in gelatinized starch urea was 999.30 g/kg, indicating that both urea forms could be almost completely digested with time.

### 3.2. Growth Performance

For the period from days 31 to 60, ADG in the PCU group was significantly higher than that in the CON group (*p* < 0.05) and the GSU group (*p* < 0.05). In addition, the ADG of the GSU group was lower (*p* < 0.05) than the CON group. The feed–weight ratio of the PCU group was significantly lower than both the CON group (*p* < 0.05) and the GSU group (*p* < 0.05) (Table 3). Similarly, over the full experimental period, ADG in the PCU group was significantly higher than that in the GSU group (*p* < 0.05) and extremely significantly higher than that in the CON group (*p* < 0.05). The feed–weight ratio of the PCU group was significantly lower than both the GSU group (*p* < 0.05) and the CON group (*p* < 0.05) during the 90 day feeding period. There was no significant difference in other growth performance indexes of each test cycle (*p* < 0.05).

### 3.3. Apparent Digestibility

The apparent digestibility of EE in the GSU group was significantly higher than that in the CON group (*p* < 0.05), with the PCU group being intermediate (Table 4). The ADF apparent digestibility in the PCU group was significantly higher than that of the CON group (*p* < 0.05), and significantly higher than that of the GSU group (*p* < 0.05). Organic matter, crude protein, and NDF apparent digestibility were not different.

### 3.4. Serum Biochemical Indexes

Among the serum biochemical indexes of blood samples collected on the 49th day, the ALT activity in the GSU group was significantly higher than that in the PCU group (*p* < 0.05). BUN, AN, TP, ALB, GLOB, AST, and TC among all groups were not different (*p* > 0.05) (Table 5). In the serum biochemical indexes of blood samples collected on the 86th day, the ALB content in the PCU group was significantly higher than that in the CON group (*p* < 0.05). The AN concentration and ALT activities in the GSU group were significantly higher than those in the CON group. The AST activities was higher in the GSU group than the PCU group. There were no significant differences in BUN, TP, GLOB, TC, and A/G among the groups.

### 3.5. Rumen Fermentation Indexes

In the fermentation indexes of rumen fluid samples, the contents of acetate and butyrate (*p* < 0.05), as well as total VFA and propionate (*p* < 0.05), in the PCU group were higher than those in the CON group (Table 6). The contents of propionate and butyrate in the GSU group were also higher than those in CON group (*p* < 0.05). The ratio of acetate to propionate in the CON group was significantly higher than that of both the PCU and GSU groups (*p* < 0.05). In addition, compared with CON group, slow-release urea decreased the content of NH3-N in the rumen (*p* < 0.05). There was no significant difference in either rumen pH (*p* > 0.05).

### 3.6. Rumen Bacterial Community

**Figure 2:**
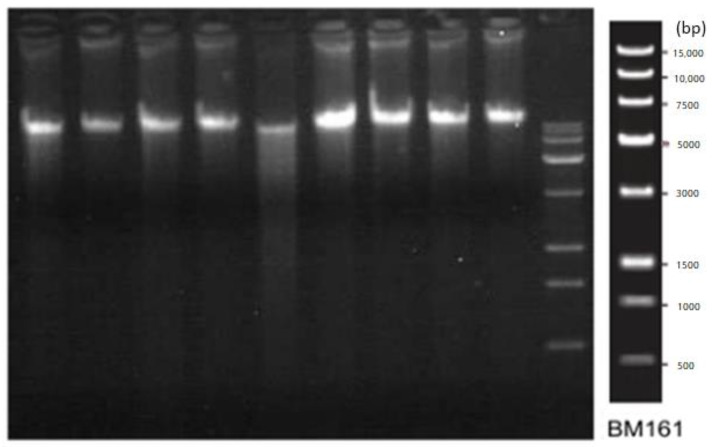
Agarose gel electrophoresis of DNA sample. From left to right, the strips are CON 1; CON 2; CON 3; PCU1; PCU 2; PCU 3; GSU 1; GSU 2; GSU 3.

The total DNA extracted from rumen fluid was detected by 1% agarose gel electrophoresis (Figure 2). The length of DNA was about 20 kb, and there was an obvious bright band, indicating that the quality of sample DNA extraction was great and the concentration was high, which could be used for subsequent detection and analysis.

### 3.7. Sample Sequence Information

In this experiment, 1,257,144 original sequences and 1,246,088 effective sequences were obtained from the three groups of samples. The average lengths of effective sequences were 420.04 bp, 419.69 bp, 420.89 bp, 420.68 bp, 417.93 bp, 420.31 bp, 421.12 bp, 421.55 bp, and 421.94 bp. The optimized proportions of high-quality sequences were 99.43%, 99.66%, 99.26%, 99.75%, 95.89%, 99.47%, 99.26%, 99.36%, and 99.77%, respectively (Table 7).

### 3.8. Diversity of Rumen Bacterial Community Structure

As presented in Table 8, the Chao1 index in the PCU group was significantly higher than that in the GSU group (*p* < 0.05). ACE index of the PCU group was greater than that in the GSU group (*p* < 0.05). The Shannon index of the PCU group was significantly higher than that of the GSU group (*p* < 0.05), indicating that the diversity of bacteria in the PCU group was higher than that in the GSU group.

### 3.9. Bacterial Community Structure Analysis Based on Gate Level

**Figure 3:**
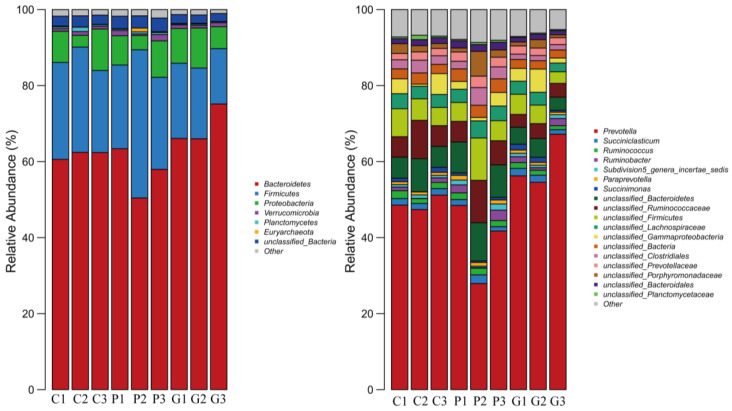
The relative abundance of bacteria at the phylum and genus levels in the CON, PCU, and GSU groups.

The dominant phyla of rumen bacteria in cattle across all three treatment groups were *Bacteroidetes*, *Firmicutes*, and *Proteobacteria* (Figure 3). The respective relative abundances for the CON group were 61.95%, 24.86%, and 7.35; for the PCU group were 57.35%, 28.37%, and 6.98%; and for the GSU group were 69.21%, 17.61%, and 8.43% (Table 9). The relative abundance of Bacteroidetes in the GSU group was significantly higher than that in the PCU group (*p* < 0.05). Compared with the CON group, the relative abundance of Firmicutes in the PCU group was 14.12% higher (*p* > 0.05), while that in the GSU group was 29.16% lower (*p* > 0.05). There was no significant difference in the relative abundance of bacterial communities at other phylum levels (*p* > 0.05).

### 3.10. Bacterial Community Structure Analysis Based on Genus Level

The proportion of *Prevotella* content in the GSU group was significantly higher than that in the PCU group (*p* < 0.05); *Paraprevotella* in the PCU group was significantly higher than that in the CON group and the GSU group (*p* < 0.05) (Table 10). There was no significant difference in the proportion of bacterial communities in other genera measured (*p* > 0.05).

### 3.11. Total DNA Integrity Results of Ruminal Fungi

**Figure 4:**
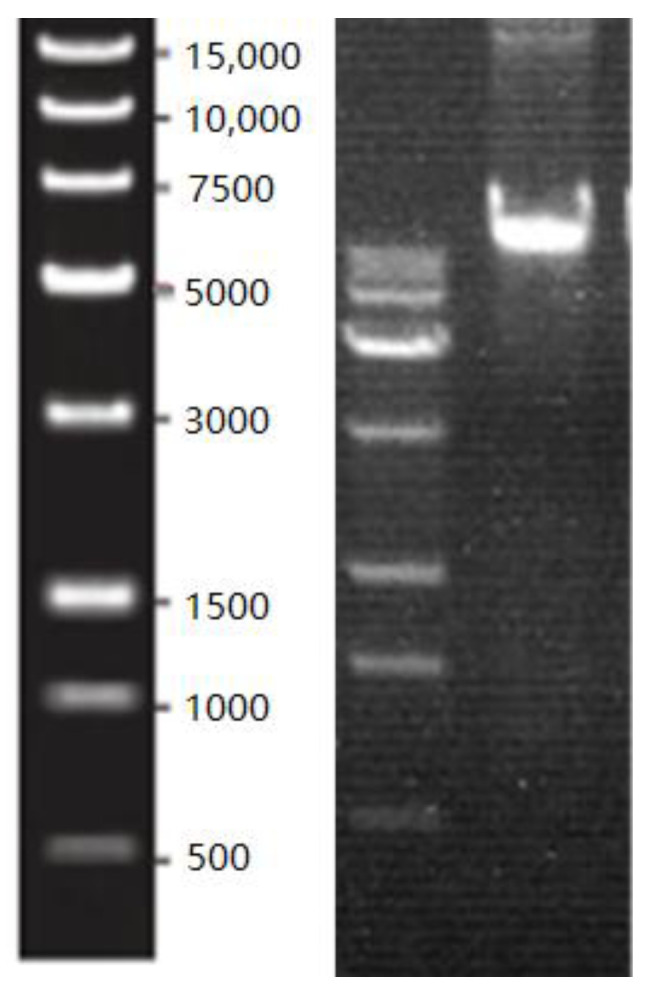
Agarose gel electrophoresis of DNA samples.

The total DNA extracted from rumen fluid was detected by 1% agarose gel electrophoresis (Figure 4). The DNA length was about 20 kb and had obvious bright bands, indicating that the quality of DNA extraction was good, and the concentration was high, which allowed for subsequent detection and analysis.

### 3.12. Sample Sequence Information

A total of 511,819 raw fungal sequences were obtained in this study. After quality control and filtering, 506,943 valid sequences were analyzed. The average lengths of effective sequences were 277.58 bp, 275.62 bp, 286.12 bp, 290.17 bp, 286.13 bp, 292.67 bp, 287.73 bp, 259.96 bp, and 220.55 bp. The optimized proportions of high-quality sequence were 99.62%, 99.88%, 99.80%, 99.77%, 95.23%, 99.67%, 99.62%, 99.89%, and 96.05%, respectively (Table 11).

### 3.13. Diversity of Ruminal Fungi Community Structure

There was no significant difference in Chao1 index, ACE index, Shannon index, and Simpson index among the groups (*p* > 0.05) (Table 12). Therefore, there was no significant difference in species richness, evenness, or diversity of fungi in the samples.

### 3.14. Fungi Community Structure Analysis Based on Gate Level

**Figure 5:**
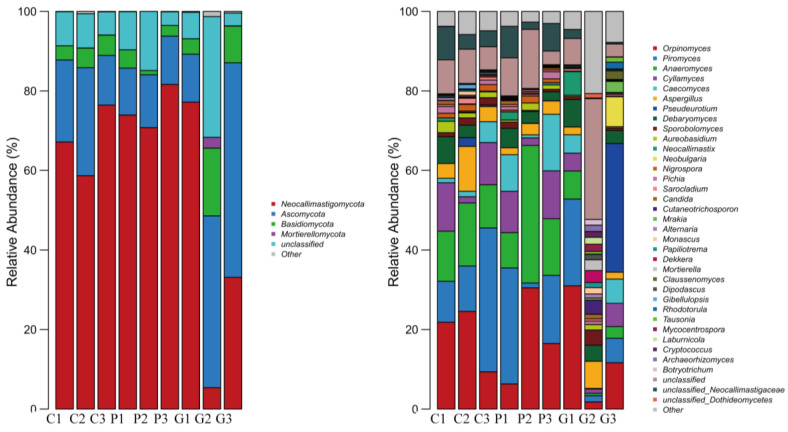
Histogram of relative abundance of fungi at the phylum and genus levels.

Four phyla of fungi were obtained by annotation of three groups of rumen microorganisms (Figure 5). The dominant ruminal fungi in each group were *Neocallimastigomycota* and *Ascomycota* (Table 13). Their relative abundances in the CON group were 67.42% and 20.09%, respectively. In the PCU group, their abundances were 75.45% and 12.40%, and in the GSU group, 38.56% and 36.40%, respectively.

### 3.15. Analysis of Fungal Community Structure Based on Genus Level

The 11 fungi genera with the highest relative abundance in the rumen across all treatments were *Orpinomyces*, *Piromyces*, *Anaeromyces*, *Cyllamyces*, *Caecomyces*, *Aspergillus*, *Pseudeurotium*, *Debaryomyce*, *Sporobolomyces*, *Aureobasidium*, and *Neobulgaria* (Figure 5 and Table 14).

## 4. Discussion

Polymer-coated urea and gelatinized starch urea are two commonly used slow-release urea sources in ruminant feed [1]. Urea that is released too quickly will not align with the microbial digestion and energy availability from other feedstuffs. At the same time, if the release rate of urea is too slow, the urea may not be fully available to the rumen microbes before passing out of the rumen, minimizing its benefits to rumen fermentation [7]. The results of this experiment showed that the gelatinized starch urea reached 93.53% release after two hours, and thus the slow-release effect was poor. Previous research with 22 gelatinized starch urea samples collected from eight sources nationwide found that the dissolution of six samples was above 90% in 2 h [14]. The release of nitrogen from the polymer-coated urea was 62.43% at the second hour and 95.10% after eight hours. This suggests slower sustained release of urea that is more likely to be fully utilized by rumen microbes.

The addition of polymer-coated urea and gelatinized starch urea to replace some protein ingredients in beef cattle diets in this study had no effect on feed intake, which is in agreement with the results of previous studies [4]. There was no significant difference in DMI, ADG, and feed-to-weight ratio of beef cattle compared with the soybean meal group when 1.1% of the polymer-coated urea was added to the diet [15]. Diets supplemented with 1.2% the slow-release urea (Agri-Nutrient Technology Group, Saudi Arabia) showed non-significant differences in DMI, ADG, and feed-to-weight ratio of beef cattle compared to the control group [16]. The differences in DMI, ADG, and feed-to-weight ratio were not significant in the Angus steers diets supplemented with 1.3% and 3.1% of the Optigen II compared to the group supplemented with 1.2% of conventional urea, but ADG and feed utilization efficiency were higher in the PCU group than in the conventional urea group [17]. It has also been shown that under isoenergetic and isonitrogenous conditions, DMI, ADG, and feed utilization efficiency were higher among all groups than in the control group when urea phosphate was added at no more than 4% to the concentrate supplement, which is consistent with the results of previous studies [18]. However, when urea phosphate was added at 8% to the concentrate supplement, DMI, ADG, and feed utilization efficiency of beef cattle were significantly lower than in the control group [18]. In this trial, polymer-coated urea and gelatinized starch urea were added at 2% and 2.5%, respectively, in this trial. The results showed that in the second month, the average daily weight gain of the PCU group were significantly higher than that of the CON group and the GSU group, and the feed-to-weight ratio was significantly lower than that of the other two groups; this might have been due to the different physiological states of the cattle at this stage. Throughout the test period, the ADG of the PCU group was significantly higher than that of the GSU group and significantly higher than that of the CON group, while the feed-to-weight ratio was significantly lower than that of the other two groups. This suggests that the best weight gain effect of polymer-coated urea may be related to its better slow-release performance, which can continue to release urea after 2h of feeding, maintain the appropriate rumen concentration of ammonia nitrogen, provide more nitrogen sources for rumen microorganisms, and increase nitrogen deposition. The results of some studies showed that the feed-to-weight ratio increased when urea was inserted at 0.8% or more [19]. For the inconsistent results of this study, the reason may be related to the structure of the diet and CP level, the test diet in the refined to concentrate-roughage ratio of 4:6, and the crude protein content of 14%, while the present test diet was refined to a concentrate-roughage ratio of about 7:3; the diet NDF content higher crude protein content was lower at 11.8%, and potentially in the case of high roughage content and low-protein diets, added urea is conducive to improving the feed conversion efficiency [20]. The daily weight gain and feed conversion efficiency in the first month of the GSU group were higher than those of the other two group, which may have been due to the different adaptation mechanisms and adaptation time of separate slow-release urea rumen microorganisms. The cattle may take a longer time to adapt to polymer-coated urea because of the microencapsulation, and the ammonia-using bacteria continue to keep growing and reproducing, which may take a longer time to adapt to; conversely, most of the urea was released in the gelatinized starch urea at the second hour, and the addition of starch in the gelatinized starch urea was more consistent with the trend of dietary ammonia release and energy–nitrogen balance, so the rumen microorganisms had a shorter adaptation time to the gelatinized starch urea.

In this study, the use of slow-release urea in the diets of cattle had no significant effect on the apparent digestibility of OM, CP, or NDF. This counters previous research where the utilization rate of low-quality forage and digestibility of DM and CP was improved by adding an appropriate amount of slow-release urea with lignin as a carrier in sheep diet [21]. Similarly, replacing a portion of the soybean meal in fattening cattle diets with slow-release urea has been shown to significantly increase the apparent digestibility of organic matter [22]. The apparent digestibility of EE and ADF in the PCU group was significantly higher than that in the no urea group and the gelatinized starch urea group in this study. The variation in effects on digestibility seen across trials may be related to the diet structure. The high forage structure diet slows down the rumen fermentation rate, and the addition of slow-release urea in the low-protein diet provides continuous ammonia. Ammonia can promote the growth and reproduction of fiber-degrading bacteria, thereby improving the apparent digestibility of EE and ADF in the diet [23].

Under normal conditions, urea in the rumen is degraded into ammonia, and rumen microorganisms use ammonia to synthesize bacterial protein. Excessive unused ammonia is absorbed by rumen wall into the blood [24]. The blood ammonia concentration is closely related to the crude protein level in the diet. If the blood ammonia concentration increases, it indicates that the urea in the diet is wasted and not fully utilized by the microbial population [25]. The results of this experiment showed no significant difference in the blood ammonia concentration between the PCU group and the CON group, indicating that the polymer-coated urea was well utilized in the rumen. This is consistent with previous studies [26]. However, in the 12th week, the blood ammonia concentration in the GSU group was significantly higher than that in the CON group, indicating that the degradation rate of gelatinized starch urea in the rumen was faster than the rumen microorganisms were able to utilize the urea, resulting in a waste of nitrogen and a lack of synchronization in protein synthesis with urea release. TP can reflect the absorption, synthesis, and metabolism of body proteins; ALB is a sensitive indicator of early nutritional status of proteins; and serum BUN can reflect nitrogen metabolism and deposition, as well as the utilization of crude protein in feed [27]. The results of this study showed that there was no significant difference in TP, GLOB, or BUN contents among groups on the 49th or 86th days, which was consistent with previous studies [28,29]. However, the ALB values in the PCU group were significantly higher than that of the no urea group on the 86th day, and the blood ammonia concentration of the PCU group was not significantly different from that of the CON group on the 86th day, indicating that the ammonia produced by the degradation of polymer-coated urea could be effectively utilized by rumen microorganisms, wherein protein metabolism was enhanced, and the blood mobilized large amounts of ALB to transport raw materials and metabolites for the synthesis of somatic tissues. AST and ALT in serum are important indicators reflecting liver function, which are involved in trans amino metabolism [30]. In this study, consistent with a previous study on the addition of different levels of urea to the diet of beef cattle, no significant difference in AST and ALT was found between the PCU group and the no urea group on the 49th and 86th days [31]. ALT in the GSU group was significantly higher than the PCU group on the 49th day and the CON group on the 86th day, which suggests that gelatinized starch urea increased the burden of liver [29]. Taken in combination, these blood indicators of nitrogen metabolism are consistent with previous studies and indicate that the addition of slow-release urea in the diet has little impact on protein metabolism in beef cattle.

Ruminal pH is the basic index for evaluating rumen fermentation. Rumen content pH is the result of the interaction between VFA in chyme and buffer salts in saliva, and the comprehensive effect of rumen epithelium on VFA absorption and outflow with chyme [32]. Appropriate rumen pH is necessary for the robust microbial activity responsible for fiber digestion in ruminants [33]. The results of this study showed that the pH values of the PCU group and the GSU group were lower than those of the CON group, but the difference was not significant. One study showed that the addition of 0.5% and 1% urea to the test diets resulted in significantly lower rumen pH compared to the control group [31]. The addition of urea caused the rumen pH to be lower than the CON group, probably because the addition of urea increased the rumen microbial activity and increased the production of VFA, which led to a decrease in rumen pH. The NH_3_-N concentration in rumen can reflect the urea decomposition by rumen microorganisms and the utilization of protein in diet, with high values indicating that the nitrogen source in diet is sufficient, and rumen microorganisms may not be fully utilized, resulting in nitrogen waste; conversely, low values indicate insufficient nitrogen availability for the cellulolytic bacteria, which reduces the activity of cellulolytic bacteria and the efficiency of fiber decomposition [34]. Different from previous studies, the results of this study showed that there was a difference in NH_3_-N concentration in each treatment group [35]. NH_3_-N in the rumen of ruminants is the main source of nitrogen for the synthesis of microbial proteins, which are the primary source of nitrogen for the synthesis of MCP [36]. Dietary structure, rumen nitrogen and carbon sources, vitamins, mineral elements, and rumen environment all affect the synthesis of rumen MCP [37]. In the present study, slow-release urea decreased the production of NH_3_-N, indicating that feeding slow-release urea may inhibit rumen MCP synthesis to some extent. VFA content and ratio have a great influence on the energy use efficiency of ruminants, and VFA concentration in the rumen is also the main factor affecting rumen pH [38]. The results of this experimental study showed that the TVFA content of the PCU group was significantly higher than that of the CON group. This result ties in well with previous studies wherein the TVFA content in the rumen was significantly higher than that in the no urea group when the addition of GSU was less than 1.5% in diet of beef cattle [31]. However, it does run contrary to the results of adding polymer-coated urea into the diet of castrated bulls without increasing the content of rumen TVFA. As with other measures, these differences may be related to the diet structure. In this study, the forage content in dry matter of TMR diet accounted for 70%, while the forage content in dry matter of TMR diet in the Pinos’s study was only 15.6%, which was a high concentrate diet [15].

The concentration of acetate in the rumen may be related to the absorption rate of VFA [39]. The molar ratio of acetate is positively correlated with the crude content in the diet, and as the activity of cellulolytic bacteria increased, the level of acetate also increased [40]. For ruminants, propionate is an important sugar-producing substance, and the improvement of propionate is conducive to improving the production performance of animals [41]. Propionate fermentation can utilize H_2_ produced by acetate and butyrate fermentation to improve feed efficiency [42]. Butyrate is a major product of rumen microbial fermentation and a precursor for the synthesis of milk fat and body fat [43]. The results of this trial showed that the content of acetate, butyrate, and propionate in the PCU group was significantly higher than that in the CON group. The content of propionate and butyrate in the GSU group was significantly higher than that in the CON group. It is suggested that the addition of slow-release urea increased the rumen VFA content and promoted carbohydrate degradation because the addition of urea may have provided continuous NH_3_-N to the cellulolytic bacteria, and the stimulation of NH_3_-N increased the viability of the cellulolytic bacteria and promoted the degradation of fiber in the feed, while the increase in acetate concentration increased the activity of the cellulolytic bacteria. The acetate/propionate ratio can reflect the utilization efficiency of energy in the rumen. With the decrease in the acetate/propionate ratio in the rumen, the feed utilization efficiency and nitrogen deposition capacity can be improved [44]. The acetate/propionate ratio reflects the energy utilization efficiency of rumen, and it also affects the microbial flora and energy utilization efficiency in the rumen [45]. The results of this study showed that the acetate/propionate ratio in the PCU group and the GSU group was significantly higher than that in the CON group. When combined, the VFA results of this trial indicate that the addition of slow-release urea likely promoted the degradation of carbohydrates by providing a continuous NH3-N supply to ruminal bacteria, while the increase in acetate concentration increased the activity of cellulolytic bacteria.

The NH3-N concentration in the rumen can reflect the urea decomposition by rumen microorganisms and the utilization of protein in the diet, with high values indicating that the nitrogen source in the diet is sufficient, and rumen microorganisms may not be fully utilized, resulting in nitrogen waste; conversely, low values indicate insufficient nitrogen availability for the cellulolytic bacteria, which reduces the activity of cellulolytic bacteria and the efficiency of fiber decomposition [34]. In the current study, the decline in the concentration of NH3-N might reflect changes in the microbiota.

Diet structure has a great influence on microbial community composition. Rumen microorganisms can quickly respond to changes in diets and adjust their community structure accordingly [46]. The sequencing data in this study showed that the dominant bacterial phyla in the rumen fluid of beef cattle were Bacteroidetes and Firmicutes, which is consistent with previous reports on beef cattle, goats, and sheep [47,48,49]. Generally, Bacteroidetes rumen bacteria are related to the degradation of non-fibrous carbohydrates, and they cannot secrete cellulase, so they are not the main functional bacteria in fiber degradation [50]. Firmicutes include Ruminal coccus, Vibrio butyricum, and other fiber-degrading bacteria, which play an important role in the process of fiber degradation [51]. This experiment found that the relative abundance of Bacteroidetes in the GSU group was significantly higher than that in PCU group, and the relative abundance of Firmicutes in the PCU group was 14.12% higher than that in the CON group. The bacterial community included *Prevotella*, *succinate*, *Butyrivibrio*, *Ruminococcus*, and a large number of unclassified genera. The relative abundance of *Prevotella* was the highest in the CON group and the PCU group. Conversely, the relative proportion of *Paraprevotella* was highest in the PCU group. *Prevotella* can degrade starch and plant cell wall polysaccharides in the rumen, but cannot degrade cellulose, which plays an important role in the degradation of protein in the rumen [52]. The differences at the genus level among the treatment groups may be related to the addition of different slow-release urea.

*Neocallimastigomycota* has been isolated from the digestive tract of cattle, goats, sheep, and horses, playing an important role in the degradation of plant fibers in the diet through both physical and enzymatic degradation [53,54]. Microorganisms of *Neocallimastigomycota* can also provide microbial proteins for hosts. *Ascomycota* is the largest group of microorganisms in the fungal community, being mainly involved in the degradation of lignin and keratin in the diet [55]. Across all three treatments, *Neocallimastigomycota*, *Ascomycota*, and *Basidiomycota* were the dominant rumen fungal phyla, which is consistent with previous reports in dairy cows and other ruminants [56]. The nitrogen sources required for fungal growth are ammonia nitrogen and amino acids, but fungi are more inclined to synthesize proteins using mixed amino acids, and thus amino acids are better than ammonia in promoting the growth of ruminal fungi [57]. This may be the reason that slow-release urea did not affect the ruminal fungi in this study. We only analyzed the fungi in the rumen fluid, but there was a significant difference between the solid phase of the rumen fluid and the fungi in the liquid phase of the rumen fluid. We speculate that this may be one of the reasons that the addition of the polymer-coated urea and gelatinized starch urea had no obvious effect on the rumen fungi. Similarly, the sampling process can lead to a large error in the results, as some fungi can enter the rumen with a gastric tube.

## 5. Conclusions

The urea release profile of polymer-coated urea was superior to that of gelatinized starch urea, which could help synchronize nitrogen release with energy metabolism in the rumen and improve microbial growth patterns. The addition of polymer-coated urea in the diet changed the composition of the rumen microbial flora. This likely translated to rumen fermentation benefits through more VFA production and higher apparent digestibility of ADF. Based on these results, in the case of high dietary crude feed content, the use of partially polymer-coated urea or gelatinized starch urea instead of protein raw materials in the diet had no adverse effect on beef cattle production performance, and the former was more beneficial to performance than the latter.

## Figures and Tables

**Table 1:** Ingredient and chemical composition of total mixed rations (as-fed basis).

Item	Diet		
	CON	PCU	GSU
Straw (kg)	2.8	2.8	2.8
Corn silage (kg)	9.0	9.0	9.0
Concentrate supplements (kg)	2.3	2.3	2.3
Total (kg)	14.1	14.1	14.1
Chemical composition (%, DM)			
Dry matter (%)	49.39	49.39	49.40
ME (MCal/kg)	2.22	2.23	2.24
Crude protein (%)	11.77	11.76	11.75
EE (%)	2.67	2.76	2.77
NDF (%)	51.38	51.44	51.29
ADF (%)	30.14	30.07	30.02

**Table 2:** Ingredient and chemical composition of concentrated feed supplement (air-dried basis).

Item	CON	PCU	GSU
Corn	20.0	30.4	30.8
Palm meal	10.0	13.0	13.0
Wheat bran	16.2	16.8	15.5
Sprayed corn husk	11.0	10.0	10.0
Soybean meal	13.0	2.0	2.0
Rapeseed meal	6.0	5.0	5.0
Sesame meal	8.0	6.0	6.4
DDGS	10.0	9.0	9.0
Polymer-coated urea	—	2.0	—
Gelatinized starch urea	—	—	2.5
Premix	5.8	5.8	5.8
Total	100.0	100.0	100.0
Chemical composition (%, DM)			
Dry matter (%)	88.45	87.43	88.45
ME (MCal/kg)	2.79	2.78	2.79
Crude protein (%)	24.76	24.76	24.74

**Table 3:** Effects of different nitrogen sources on the growth performance of beef cattle.

Item	CON	PCU	GSU
Initial body weight, kg	321.91 ± 44.40	323.97 ± 46.96	322.24 ± 46.51
30 day body weight, kg	347.76 ± 46.07	350.84 ± 47.84	349.87 ± 48.11
60 day body weight, kg	370.83 ± 46.21	376.89 ± 50.52	370.60 ± 48.85
90 day body weight, kg	384.59 ± 44.69	392.80 ± 48.97	386.29 ± 49.16
Average daily gain (ADG), kg d^−1^			
0–30 days	0.86 ± 0.20	0.90 ± 0.25	0.92 ± 0.23
31–60 days	0.77 ± 0.20 ^b^	0.87 ± 0.20 ^a^	0.69 ± 0.19 ^c^
61–90 days	0.46 ± 0.23	0.53 ± 0.22	0.52 ± 0.22
0–90 days	0.70 ± 0.09 ^b^	0.76 ± 0.14 ^a^	0.71 ± 0.13 ^b^
Dry matter intake (DMI), kg			
0–30 days	6.76 ± 0.38	6.69 ± 0.33	6.77 ± 0.29
31–60 days	6.91 ± 0.20	6.89 ± 0.19	6.95 ± 0.18
61–90 days	7.03 ± 0.20	6.93 ± 0.13	7.02 ± 0.26
0–90 days	6.90 ± 0.29	6.84 ± 0.25	6.91 ± 0.27
Feed-weight ratio (DMI/ADG)			
0–30 days	8.54 ± 3.67	8.13 ± 2.75	7.89 ± 2.41
31–60 days	9.65 ± 3.22 ^b^	8.72 ± 4.43 ^b^	11.37 ± 5.73 ^a^
61–90 days	18.57 ± 20.05	15.87 ± 9.36	12.18 ± 28.23
0–90 days	10.09 ± 1.39 ^a^	9.33 ± 2.38 ^b^	10.05 ± 2.00 ^a^

^a,b,c^ Mean values within a row with different letters differ at *p* < 0.05.

**Table 4:** Effects of different nitrogen sources on the apparent digestibility of nutrients in cattle.

Item	CON	PCU	GSU
Organic matter (OM)	69.27 ± 0.13	69.13 ± 1.01	69.31 ± 0.23
Crude protein (CP)	66.98 ± 2.18	66.93 ± 0.98	67.19 ± 1.67
Ether extract (EE)	85.84 ± 0.59 ^b^	87.07 ± 1.42 ^ab^	87.70 ± 0.36 ^a^
Neutral detergent fiber (NDF)	60.79 ± 2.25	63.22 ± 2.88	63.70 ± 1.90
Acid detergent fiber (ADF)	56.74 ± 1.25 ^b^	57.79 ± 1.16 ^a^	55.36 ± 1.12 ^b^

^a,b^ Mean values within a row with different letters differ at *p* < 0.05.

**Table 5:** Effects of different slow-release urea on serum biochemical indexes in beef cattle.

Item	CON	PCU	GSU
49th day			
BUN (mmol/L)	3.73 ± 0.58	3.53 ± 0.50	3.72 ± 0.34
AN (μmol/L)	91.88 ± 11.43	97.25 ± 18.90	99.38 ± 12.11
TP (g/L)	61.10 ± 3.41	62.00 ± 0.98	62.20 ± 2.89
ALB (g/L)	35.54 ± 1.15	35.24 ± 2.18	36.05 ± 1.36
GLOB (g/L)	25.56 ± 2.90	25.61 ± 3.06	26.15 ± 2.76
A/G	1.41 ± 0.17	1.39 ± 0.19	1.39 ± 0.16
ALT (U/L)	22.19 ± 2.05 ^ab^	21.48 ± 2.11 ^b^	24.23 ± 3.75 ^a^
AST (U/L)	50.18 ± 4.10	46.78 ± 5.44	50.56 ± 4.63
TC (mmol/L)	3.63 ± 0.70	3.74 ± 0.46	3.64 ± 0.51
86th day			
BUN (mmol/L)	3.64 ± 0.48	3.66 ± 0.41	3.68 ± 0.22
AN (μmol/L)	86.50 ± 17.31 ^b^	95.50 ± 14.47 ^ab^	105.44 ± 14.20 ^a^
TP (g/L)	59.54 ± 3.45	61.41 ± 2.99	59.80 ± 2.33
ALB (g/L)	33.29 ± 1.81 ^b^	35.02 ± 1.42 ^a^	34.05 ± 1.05 ^ab^
GLOB (g/L)	27.26 ± 1.47	26.39 ± 2.68	25.54 ± 1.34
A/G	1.28 ± 0.13	1.34 ± 0.16	1.31 ± 0.11
ALT (U/L)	21.50 ± 1.66 ^b^	21.87 ± 1.55 ^ab^	23.72 ± 2.98 ^a^
AST (U/L)	48.14 ± 6.73 ^ab^	44.68 ± 5.28 ^b^	51.42 ± 7.10 ^a^
TC (mmol/L)	3.36 ± 0.59	3.46 ± 0.42	3.25 ± 0.42

TP = total protein, ALB = albumin, GLOB = globulin, ALT = alanine aminotransferase, AST = aspartate aminotransferase, TC = total cholesterol, BUN = blood urea-N, AN = ammonia, A/G = ratio between albumin and globulin. ^a,b^ Mean values within a row with different letters differ at *p* < 0.05.

**Table 6:** Effects of different slow-release urea on rumen fermentation index in beef cattle.

Item	CON	PCU	GSU
Rumen pH	7.04 ± 0.20	7.01 ± 0.10	7.00 ± 0.13
NH3-N (mg/dL)	14.69 ± 0.24 ^c^	14.33 ± 0.12 ^b^	13.95 ± 0.20 ^a^
Total VFA (mmol/L)	71.54 ± 12.02 ^b^	82.53 ± 4.97 ^a^	78.21 ± 5.67 ^ab^
Acetate (mmol/L)	41.47 ± 7.56 ^b^	46.95 ± 2.96 ^a^	43.86 ± 4.38 ^ab^
Propionate (mmol/L)	18.50 ± 3.74 ^b^	22.26 ± 1.59 ^a^	21.00 ± 1.89 ^a^
Butyrate (mmol/L)	11.56 ± 1.40 ^b^	13.32 ± 1.42 ^a^	13.35 ± 2.02 ^a^
Acetate/propionate	2.26 ± 0.20 ^a^	2.11 ± 0.10 ^b^	2.09 ± 0.91 ^b^

^a,b,c^ Mean values within a row with different letters differ at *p* < 0.05.

**Table 7:** Sequencing information of ruminal bacteria.

Sample ID	Raw Reads	Effect Reads	Mean Length	Effective Sequence Ratio%
CON1	137,422	136,632	420.04	99.43
CON2	121,124	120,716	419.69	99.66
CON3	130,021	129,065	420.89	99.26
PCU1	144,836	144,470	420.68	99.75
PCU2	132,545	127,094	417.93	95.89
PCU3	138,826	138,093	420.31	99.47
GSU1	145,376	144,299	421.12	99.26
GSU2	140,075	139,183	421.55	99.36
GSU3	166,919	166,536	421.94	99.77

**Table 8:** Alpha diversity index statistical table of ruminal bacteria.

Item	CON	PCU	GSU
Chao1_index	2060.44 ± 44.57 ^ab^	2091.41 ± 55.39 ^a^	1977.89 ± 14.60 ^b^
ACE index	2043.16 ± 22.14 ^ab^	2078.21 ± 39.74 ^a^	1973.46 ± 17.62 ^b^
Shannon index	5.63 ± 0.06 ^ab^	5.86 ± 0.10 ^a^	5.33 ± 0.19 ^b^
Simpson index	0.01 ± 0.00	0.01 ± 0.00	0.02 ± 0.00
Coverage %	99.67 ± 0.05	99.72 ± 0.08	99.73 ± 0.04

^a,b^ Mean values within a row with different letters differing at *p* < 0.05.

**Table 9:** Statistical table of the proportion of the ruminal dominant bacteria level of samples.

Item	CON	PCU	GSU
*Bacteroidetes*	61.95 ± 1.03 ^ab^	57.35 ± 6.52 ^b^	69.21 ± 5.27 ^a^
*Firmicutes*	24.86 ± 3.08	28.37 ± 9.24	17.61 ± 2.74
*Proteobacteria*	7.35 ± 3.96	6.98 ± 2.97	8.43 ± 2.45
*Verrucomicrobia*	0.80 ± 0.16	1.15 ± 0.66	0.89 ± 0.18
*Planctomycetes*	0.66 ± 0.42	0.47 ± 0.10	0.28 ± 0.03
*Euryarchaeota*	0.12 ± 0.11	0.45 ± 0.48	0.12 ± 0.86

^a,b^ Mean values within a row with different letters differing at *p* < 0.05.

**Table 10:** Statistical table of proportion of ruminal dominant bacteria in samples.

Item	CON	PCU	GSU
*Prevotella*	49.04 ± 1.94 ^ab^	39.37 ± 10.48 ^b^	59.34 ± 6.87 ^a^
*Succiniclasticum*	1.68 ± 0.06	1.66 ± 0.58	1.67 ± 0.42
*Ruminococcus*	1.66 ± 0.40	1.72 ± 0.08	1.30 ± 0.24
*Ruminobacter*	0.73 ± 0.60	1.66 ± 1.35	1.36 ± 0.63
*Subdivision5_genera_incertae_sedis*	0.74 ± 0.14	1.09 ± 0.65	0.85 ± 0.18
*Paraprevotella*	0.78 ± 0.23 ^b^	1.09 ± 0.14 ^a^	0.76 ± 0.96 ^b^
*Succinimonas*	0.76 ± 0.65	0.59 ± 0.16	1.13 ± 0.55

^a,b^ Mean values within a row with different letters differing at *p* < 0.05.

**Table 11:** Sequencing information of ruminal fungi.

Sample ID	Raw Reads	Effect Reads	Mean Len	Effect Reads Ratio%
CON1	41,825	41,666	277.58	99.62
CON2	69,116	69,031	275.62	99.88
CON3	60,506	60,384	286.12	99.80
PCU1	49,506	49,391	290.17	99.77
PCU2	39,492	39,189	286.13	95.23
PCU3	45,498	45,350	292.67	99.67
GSU1	46,290	46,116	287.73	99.62
GSU2	65,615	65,545	259.96	99.89
GSU3	93,971	90,262	220.55	96.05

**Table 12:** Alpha diversity index statistical table of ruminal fungi.

Item	CON	PCU	GSU
Chao1_index	433.89 ± 34.50	484.27 ± 109.50	609.86 ± 144.27
ACE index	434.55 ± 37.08	479.06 ± 108.76	605.47 ± 146.49
Shannon index	3.75 ± 0.23	3.81 ± 0.15	3.97 ± 0.98
Simpson index	0.07 ± 0.04	0.05 ± 0.02	0.08 ± 0.06
Coverage %	99.92 ± 0.06	99.87 ± 0.06	99.92 ± 0.06

**Table 13:** The statistical table of the proportion of the level of dominant fungi in the rumen of the sample.

Item	CON	PCU	GSU
*Neocallimastigomycota*	67.42 ± 8.89	75.45 ± 5.59	38.56 ± 36.21
*Ascomycota*	20.09 ± 7.36	12.40 ± 0.78	36.40 ± 21.76
*Basidiomycota*	4.53 ± 0.84	2.79 ± 1.77	10.05 ± 6.62
*Mortierellomycota*	0.03 ± 0.02	0.00 ± 0.00	0.93 ± 1.55
*Unclassified*	7.67 ± 1.60	9.29 ± 5.73	13.41 ± 14.78
Other	0.26 ± 0.29	0.06 ± 0.05	0.65 ± 0.57

**Table 14:** Statistical table of the proportion of ruminal dominant fungi in the samples.

Item	CON	PCU	GSU
*Orpinomyces*	18.57 ± 8.08	17.76 ± 12.14	14.82 ± 14.86
*Piromyces*	19.32 ± 14.62	15.84 ± 14.02	9.84 ± 10.62
*Anaeromyces*	13.10 ± 2.56	19.24 ± 13.57	3.51 ± 3.26
*Cyllamyces*	8.11 ± 5.73	8.09 ± 5.45	3.84 ± 2.46
*Caecomyces*	2.59 ± 2.32	8.08 ± 6.81	3.62 ± 3.10
*Aspergillus*	6.27 ± 4.34	2.66 ± 0.81	3.50 ± 2.88
*Pseudeurotium*	0.74 ± 1.28	0.04 ± 0.01	10.80 ± 18.69
*Debaryomyces*	3.42 ± 3.19	3.35 ± 1.33	4.74 ± 1.92
*Sporobolomyces*	1.56 ± 0.48	0.82 ± 0.66	1.71 ± 1.84
*Aureobasidium*	1.86 ± 0.89	1.16 ± 0.67	0.66 ± 0.58
*Neocallimastix*	0.40 ± 0.39	0.88 ± 1.06	2.00 ± 3.41

## Data Availability

Due to restrictions, data are available upon request.
